# Supplementary material for: Unraveling the optical shape of snow
Source: Nat Commun. 2023 Jul 7;14:3955. doi: 10.1038/s41467-023-39671-3 (PMC10329009; doi:10.1038/s41467-023-39671-3)
Supplement: Supplementary file 1 — Supplementary information [file 41467_2023_39671_MOESM1_ESM.pdf]

Supplementary information

Alvaro Robledano[1,2*], Ghislain Picard[1], Marie Dumont[2], Frédéric Flin[2], Laurent Arnaud[1] and Quentin Libois[3]

[1]Univ. Grenoble Alpes, CNRS, INRAE, IRD, Grenoble INP, IGE, 38000, Grenoble, France.
[2]Univ. Grenoble Alpes, Université de Toulouse, Météo-France, CNRS, CNRM, Centre d'Etudes de la Neige, 38000, Grenoble, France.
[3]CNRM, Université de Toulouse, Météo-France, CNRS, 31000, Toulouse, France.

*Corresponding author(s). E-mail(s): alvaro.robledano-perez@univ-grenoble-alpes.fr;
Contributing authors: ghislain.picard@univ-grenoble-alpes.fr; marie.dumont@meteo.fr; frederic.flin@meteo.fr; laurent.arnaud@univ-grenoble-alpes.fr; quentin.libois@meteo.fr;

Supplementary information to the manuscript entitled 'Unraveling the optical shape of snow' by A. Robledano, G. Picard, M. Dumont, F. Flin, L. Arnaud and Q. Libois, submitted as an Article to Nature Communications. The following includes six figures, two tables and three methods items.

1 Supplementary Figures

Supplementary Figure 1

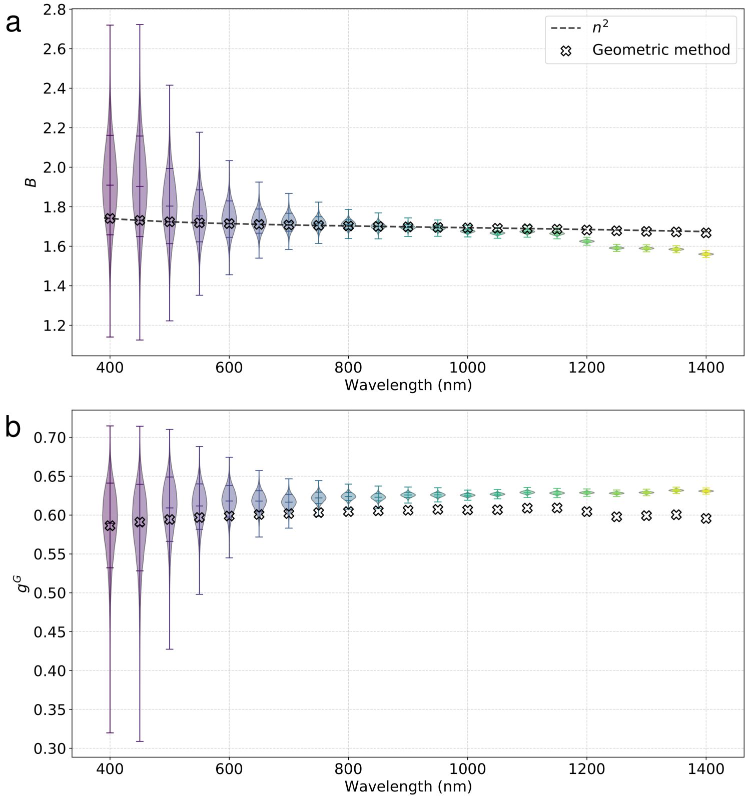

Fig. 1 Estimation of the optical shape parameters with the macroscopic method for the I23 (RG) sample. a Model estimations of the absorption enhancement parameter B represented as probability distributions, with the lines in each violin plot corresponding to the extrema, the mean, and the 10th and 90th-percentile of the resulting distributions. The geometric method estimation is also displayed. **b** Model estimations of the geometric asymmetry parameter g^G represented as probability distributions.

Supplementary Figure 2

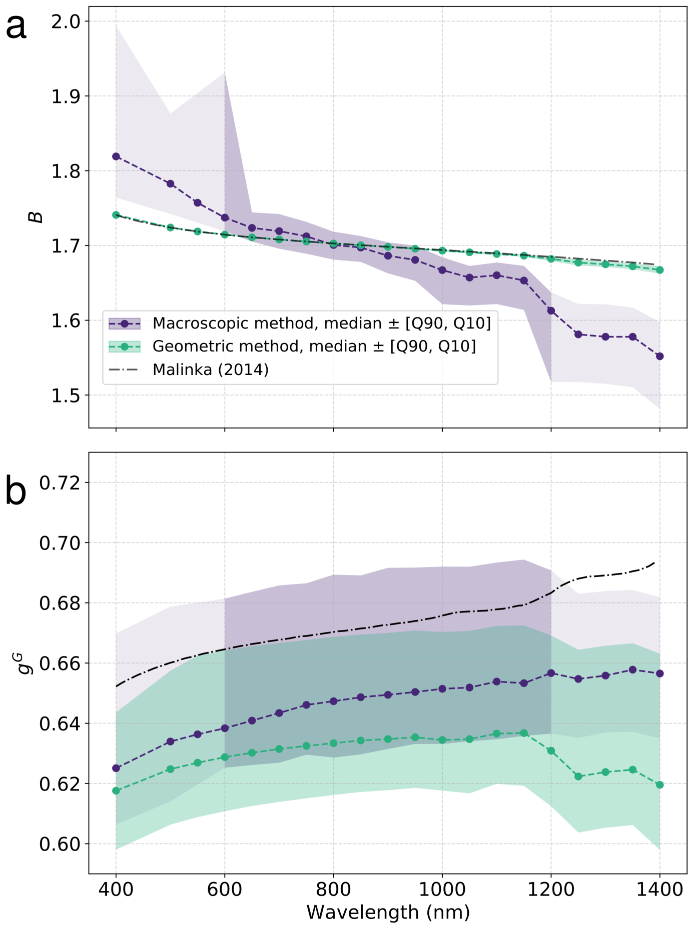

Fig. 2 Spectral variations of the optical shape parameters. a Absorption enhancement parameter B. **b** Geometric asymmetry parameter g^G. In all panels, both retrieval methods are shown, and all snow microstructure images are considered. The Malinka estimations for the two-phase random medium are included. The lower and upper limits of the envelope wrapping the median value represent the 10th and the 90th-percentile of the estimates for each wavelength. Note that the B values for the two-phase random medium are virtually equivalent to those estimated with the geometric method and equal to n^2. Note also that the macroscopic method is less accurate below 600 nm and above 1200 nm (see Method limitations). This is the reason why the envelope is more transparent for the shortest and longest wavelengths.

Supplementary Figure 3

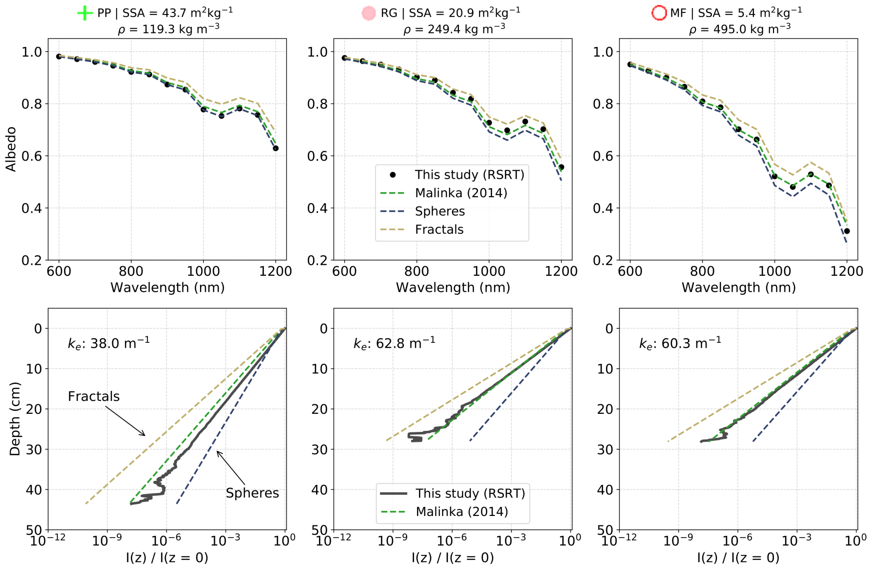

Fig. 3 Snow albedo and flux profile (I(z)). Spectral variations of snow albedo (top) and flux profile (I(z)) (bottom) at $\lambda = 900\,\mathrm{nm}$ of three different snow samples. SSA is the specific surface area, i.e. the total surface area of the air-ice interface per unit of mass, and ρ is the sample density. Spherical and fractal scenarios are computed using the AART theory [1–4] with the corresponding values of the optical shape parameters B and g^G.

Supplementary Figure 4

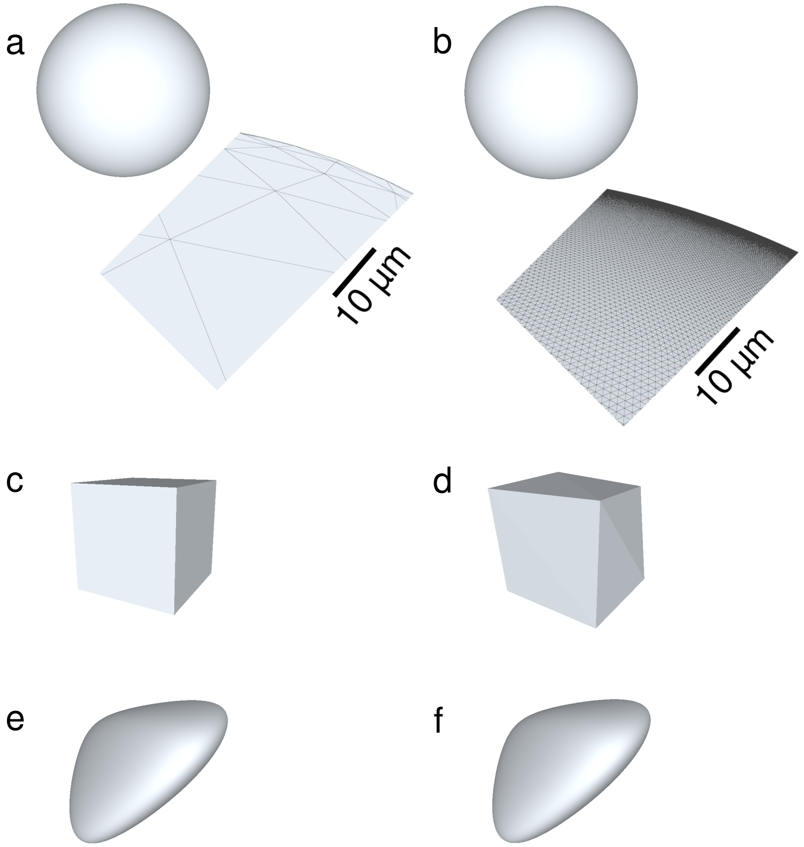

Fig. 4 Generated geometric shapes. a Almost perfect sphere, composed of ≈ 5.2 million triangular facets. **b** Deteriorated sphere, composed of ≈ 5000 triangular facets. **c** Perfect cube. **d** Spoiled cube, after translating in space each of the corners. **e** Analytical convex shape, composed of ≈ 7.1 million triangular facets. **f** Analytical convex shape, composed of ≈ 50k triangular facets. The differences between (a) and (b), and (e) and (f) are subtle and difficult to appreciate. A small part of the spheres is enlarged and shown to appreciate in detail such differences, as they have profound implications for optical properties, as shown in the main text.

Supplementary Figure 5

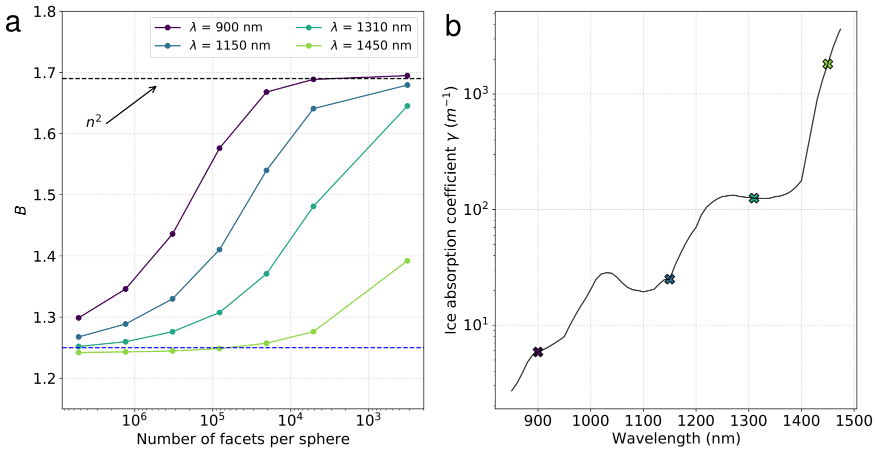

Fig. 5 Absorption enhancement parameter of spheres with an increasing absorption. a Variations of the absorption enhancement parameter B of a gradually deformed sphere when ice absorption is increased (longer wavelengths). **b** Ice absorption coefficient in the near-infrared spectral region. The crosses correspond to the wavelengths shown in the panel (a).

Supplementary Figure 6

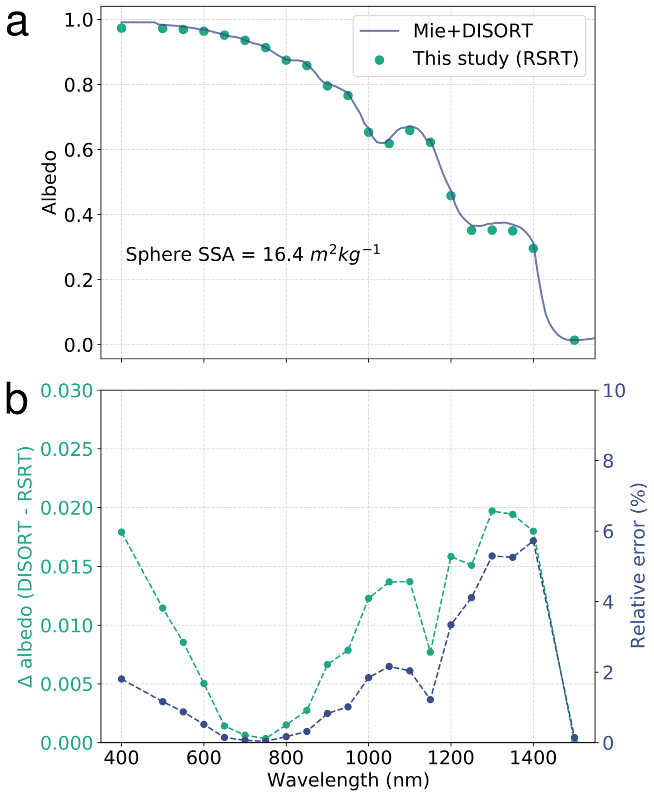

Fig. 6 Validation of the RSRT model against a Mie+DISORT model. **a** Snow spectral albedo of monodisperse spherical particles with specific surface area (SSA) of 16.4 m^2kg^{-1}. **b** Albedo differences and relative error between the RSRT model and a Mie+DISORT model.

Supplementary Tables

Supplementary Table 1

Table 1 Description of the 3D snow microstructure images dataset. Resolution corresponds to the X-ray tomography imaging pixel size, and snow properties (density and specific surface area (SSA)) were computed directly over the mesh with the trimesh Python package [5]. The snow samples were either collected in the field (French Alps) or come from controlled cold-room experiments. In the latter case, the initial sample was most of the time recent alpine snow. More details on the snow sampling and characterization are available at each of the correspondent studies.

Name	Snow type	Resolution (μm)	Density (kg m^{-3})	SSA (m^2 kg^{-1})	Study	Origin
01iso	PP	4.91	94.8	58.5	F04 [6]	Field (French Alps)
03iso	PP/DF	4.91	119.3	43.7	F04	Cold-room exp.
05iso	DF	4.91	150.2	35.5	F04	Cold-room exp.
08iso	DF/RG	4.91	141.0	31.6	F04	Cold-room exp.
15iso	DF/RG	4.91	170.0	25.4	F04	Cold-room exp.
19iso	RG	4.91	189.9	21.8	F04	Cold-room exp.
21iso	RG	4.91	194.6	21.3	F04	Cold-room exp.
23iso	RG	4.91	260.8	19.2	F04	Cold-room exp.
I08	DF/RG	4.91	144.3	31.8	F04	Cold-room exp.
I23	RG	4.91	249.4	20.9	F04	Cold-room exp.
E2bis	DH/FC	4.91	234.8	16.9	F11 [7]	Cold-room exp.
INH5	MF	8.59	494.9	5.4	F11	Cold-room exp.
NH5G	MF	9.46	471.5	4.6	F11	Cold-room exp.
IP04	DF	8.59	154.5	27.1	C12 [8]	Field (French Alps)
IP06	RG	6.16	353.3	23.8	C12	Field (French Alps)
IP08	RG	8.55	376.9	16.5	C12	Field (French Alps)
IP10	RG	6.10	393.0	12.6	C12	Field (French Alps)
IP15	RG	6.16	312.6	18.2	C12	Field (French Alps)
7G9	DH	9.67	316.9	14.1	C14 [9]	Cold-room exp.
1600sup800	RG	7.43	326.6	17.3	W15 [10]	Cold-room exp.
E17	DF/RG	7.25	145.1	21.6	D21 [11]	Cold-room exp.
G1-10	DF/RG	9.71	132.7	22.8	D21	Cold-room exp.
I17	DF/RG	7.25	142.0	21.4	D21	Cold-room exp.
I1-10	DF/RG	9.71	117.9	22.3	D21	Cold-room exp.
J17	DF/RG	7.30	149.1	21.9	D21	Cold-room exp.
TG1	FC/DH	7.06	281.9	18.1	D21	Cold-room exp.
TG2	FC/DH	7.03	245.8	19.8	D21	Cold-room exp.
TG3	FC/DH	7.06	343.4	16.1	D21	Cold-room exp.
TG4	FC/DH	7.06	352.4	17.4	D21	Cold-room exp.
TG5-10	FC/DH	11.65	262.9	15.4	D21	Cold-room exp.
TG5-5	FC/DH	5.83	254.8	17.5	D21	Cold-room exp.
TG5-7	FC/DH	7.07	253.0	17.4	D21	Cold-room exp.
TG8	FC/DH	7.07	320.8	16.7	D21	Cold-room exp.

Supplementary Table 2

Table 2 Optical shape parameters of geometric shapes. Values of B and g^G for a diverse set of geometric shapes, computed with ray-tracing models in previous studies. The aspect ratio for hexagonal plates, cuboids and cylinders is the ratio of the height to, respectively, the length of the hexagonal side, the length of the square side and the radius of the circle. For the spheroid, it is the ratio of the largest semi-axis to the shortest one.

Geometric shape	Aspect ratio	B	g^G	Study
Sphere	1	1.25	0.79	Libois et al. (2013) [2]
Spheroid	2	1.43	0.71	Kokhanovsky and Macke (1997) [12]
Hexagonal plate	1	1.60	0.57	Kokhanovsky and Macke (1997)
Hexagonal plate	2	1.60	0.52	Kokhanovsky and Macke (1997)
Cube	1	1.56	0.54	Libois et al. (2013)
Cuboid	2	1.58	0.59	Libois et al. (2013)
Cylinder	1	1.44	0.63	Libois et al. (2013)
Cylinder	2	1.46	0.69	Libois et al. (2013)
Fractal	-	1.84	0.50	Kokhanovsky and Zege (2004) [1]

Supplementary Methods

Supplementary Methods 1: B and g^G for the two-phase random medium

For the sake of completeness, we show here the expressions of the optical shape parameters B and g^G for the two-phase random medium, which are actually derived in [13]. Considering that in such medium, along a straight line, the positions of consecutive air-ice interfaces follows a Markov process, it was shown that $B = n^2$ in the limit of low absorption (see Eqs. 9 and 25 in [13]).

The geometric asymmetry parameter g^G is analytically derived in the same study (Eq. 60) as:

$$g^G = \frac{1}{\omega_0}\left(r_1 + \frac{1}{n^2}\frac{t_1^2 L(\alpha)}{1 - r_1^{\text{in}} L(\alpha)}\right) \tag{1}$$

where ω_0 is the single-scattering albedo (named photon survival probability in [13] – Eq. 56), $L(\alpha)$ is the Laplace transform of the chord length distribution of the medium (Eq. 21), and where r_1, t_1 and r_1^{in} are given in an analytic way in Eqs. 29, 42 and 49 in [13].

The value of these parameters for the two-phase random medium are wavelength-dependent via the ice refractive index n, and their median values across the visible and NIR spectral range (400 - 1400 nm) are $B = 1.69$ and $g^G = 0.67$.

Supplementary Methods 2: Analytical convex shape generation

The analytical convex shape explored in this study is generated with the following parametric equations:

$$\begin{cases} x &= x'(2 - z) \\ y &= y'(2 + z) \\ z &= \cos\theta \end{cases}$$

where x' and y' are defined as:

$$x' = \cos\phi \cdot \sin\theta$$
$$y' = \sin\phi \cdot \sin\theta$$

with the polar angle $\theta \in [0, \pi]$ and the azimuthal angle $\phi \in [0, 2\pi]$.

Supplementary Methods 3: Fresnel's law of reflectance

When the ray path intersects an ice-air interface, a decision between reflection and refraction (and therefore change of direction) may be done. This choice is random and depends mainly on a probabilistic interpretation of the Fresnel coefficients, defined by:

$$\mathrm{R_s} = \frac{\frac{n_1}{n_2}\cos\theta_i - \sqrt{1-\left(\frac{n_1}{n_2}\right)^2(1-\cos^2\theta_i)}}{\frac{n_1}{n_2}\cos\theta_i + \sqrt{1-\left(\frac{n_1}{n_2}\right)^2(1-\cos^2{\theta_i}^2)}}$$

$$\mathrm{R_p} = \frac{\cos\theta_i - \frac{n_1}{n_2}\sqrt{1-\left(\frac{n_1}{n_2}\right)^2(1-\cos^2\theta_i)}}{\cos\theta_i + \frac{n_1}{n_2}\sqrt{1-\left(\frac{n_1}{n_2}\right)^2(1-\cos^2\theta_i)}}$$

where n_i is the refractive index of media 1 and 2 (i.e. $i = 1, 2$) and θ_i is the angle of incidence between the incoming ray $\mathbf{v}_i$ and the normal vector of the interface $\mathbf{v}_n$ (oriented towards the medium 1). The total reflected energy is then computed as:

$$R = \frac{1}{2}\left(R_\mathrm{s}^2 + R_\mathrm{p}^2\right) \tag{2}$$

and therefore the transmitted energy would simply be $T = 1-R$. Here, this deterministic interpretation is treated with a Monte Carlo approach, where a random number is drawn and compared to R. If inferior, the ray encounters a reflection, otherwise a refraction. In both cases, the ray carries all the incident energy. In case of reflection, the ray outgoing direction $\mathbf{v}_o$ is defined by:

$$\mathbf{v}_o = \mathbf{v}_i + 2\cos\theta_i \mathbf{v}_n \tag{3}$$

and in case of refraction:

$$\mathbf{v}_o = \frac{n_1}{n_2}\mathbf{v}_i + \left(\frac{n_1}{n_2}\cos\theta_i - \sqrt{1-\left(\frac{n_1}{n_2}\right)^2(1-\cos^2\theta_i)}\right)\mathbf{v}_n \tag{4}$$

Supplementary References

[1] Kokhanovsky, A. A. & Zege, E. P. Scattering optics of snow. *Appl. Opt.* **43** (7), 1589–1602 (2004). https://doi.org/10.1364/AO.43.001589 .

[2] Libois, Q. *et al.* Influence of grain shape on light penetration in snow. *Cryosphere* **7** (6), 1803–1818 (2013). https://doi.org/10.5194/tc-7-1803-2013 .

[3] ghislainp. ghislainp/snowoptics: Tc paper, tc_paper (2020). https://doi.org/10.5281/zenodo.3742138.

[4] ghislainp. ghislainp/tartes: v1.1.0 (2022). https://doi.org/10.5281/zenodo.7031229.

[5] Dawson-Haggerty, M. trimesh, 3.2.0 (2019). https://trimsh.org/.

[6] Flin, F., Brzoska, J. B., Lesaffre, B., Coléou, C. & Pieritz, R. A. Three-dimensional geometric measurements of snow microstructural evolution under isothermal conditions. *Ann. Glaciol.* **38** (1), 39–44 (2004). https://doi.org/10.3189/172756404781814942 .

[7] Flin, F. *et al.* Furukawa, Y. (ed.) *On the computations of specific surface area and specific grain contact area from snow 3D images.* (ed.Furukawa, Y.) *Proceedings of the 12th International Conference on the Physics and Chemistry (PCI 2010) of Ice held at Sapporo, Japan, on 5-10 September 2010*, 321–328 (Hokkaido University Press, Sapporo, Japan, 2011).

[8] Calonne, N. *et al.* 3-D image-based numerical computations of snow permeability: links to specific surface area, density, and microstructural anisotropy. *Cryosphere* **6** (5), 939–951 (2012). https://doi.org/10.5194/tc-6-939-2012 .

[9] Calonne, N., Flin, F., Geindreau, C., Lesaffre, B. & Rolland du Roscoat, S. Study of a temperature gradient metamorphism of snow from 3-d images: Time evolution of microstructures, physical properties and their associated anisotropy. *Cryosphere* **8** (6), 2255–2274 (2014). https://doi.org/10.5194/tc-8-2255-2014 .

[10] Wautier, A., Geindreau, C. & Flin, F. Linking snow microstructure to its macroscopic elastic stiffness tensor: A numerical homogenization method and its application to 3-D images from X-ray tomography. *Geophys. Res. Lett.* **42** (19), 8031–8041 (2015). https://doi.org/10.1002/2015GL065227 .

[11] Dumont, M. *et al.* Experimental and model-based investigation of the links between snow bidirectional reflectance and snow microstructure. *Cryosphere* **15** (8), 3921–3948 (2021). https://doi.org/10.5194/tc-15-3921-2021 .

[12] Kokhanovsky, A. A. & Macke, A. Integral light-scattering and absorption characteristics of large, nonspherical particles. *Appl. Opt.* **36** (33), 8785–8790 (1997). https://doi.org/10.1364/AO.36.008785 .

[13] Malinka, A. V. Light scattering in porous materials: Geometrical optics and stereological approach. *J. Quant. Spectrosc. Radiat. Transfer* **141**, 14—23 (2014). https://doi.org/10.1016/j.jqsrt.2014.02.022 .
